# Influence of crosslinking density on the mechanical and thermal properties of plant oil-based epoxy resin†

**DOI:** 10.1039/d2ra04206a

**Published:** 2022-08-16

**Authors:** Jinrui Huang, Pan Fu, Wenbin Li, Laihui Xiao, Jie Chen, Xiaoan Nie

**Affiliations:** Institute of Ecological Conservation and Restoration, Chinese Academy of Forestry Beijing 100091 China huangjinruiahu@126.com jrhuang@caf.ac.cn; Key Laboratory of Biomass Energy and Material, Jiangsu Province, Jiangsu Co-Innovation Center of Efficient Processing and Utilization of Forest Resources, Key Laboratory of Chemical Engineering of Forest Products, National Forestry and Grassland Administration, National Engineering Research Center for Low-Carbon Processing and Utilization of Forest Biomass, Institute of Chemical Industry of Forest Products, Chinese Academy of Forestry Nanjing 210042 Jiangsu Province China niexiaoan@icifp.cn

## Abstract

Plant oil-based epoxy resins are of great interest due to their ecological and economic necessity. Previous studies suggested that the crosslinking density had a considerable influence on the mechanical and thermal properties of plant oil-based epoxy resins. However, so far, the relationship between the crosslinking density and the thermo-mechanical properties of plant oil-based epoxy resins is not clear. To address this issue, model tung oil-based epoxy resins with different crosslinking densities were fabricated to investigate the influence of crosslinking density on the mechanical and thermal properties of tung oil-based epoxy resins. Results show that the tensile strength, Young's modulus, and glass transition temperature are linearly increased with increasing crosslinking density. The elongation at break and tensile toughness show nonlinear downward trends as the crosslinking density increases. The elongation at break decreases gently at first, then dramatically, and finally slowly as the crosslinking density increases. The tensile toughness declines sharply at first and then slowly with increasing crosslinking density. The relationship between the thermostability and the crosslinking density is complex, because the thermostability is determined by both the molecular structure of the curing system and the crosslinking density. These results provide some information for designing plant oil-based epoxy resins according to the requirements of their applications.

## Abstract 2

Plant oil-based epoxy resins are of great interest due to their ecological and economic necessity. In this study, the relationship between the crosslinking density and the thermo-mechanical properties of tung oil-based epoxy resins was established.

## Graphical abstract

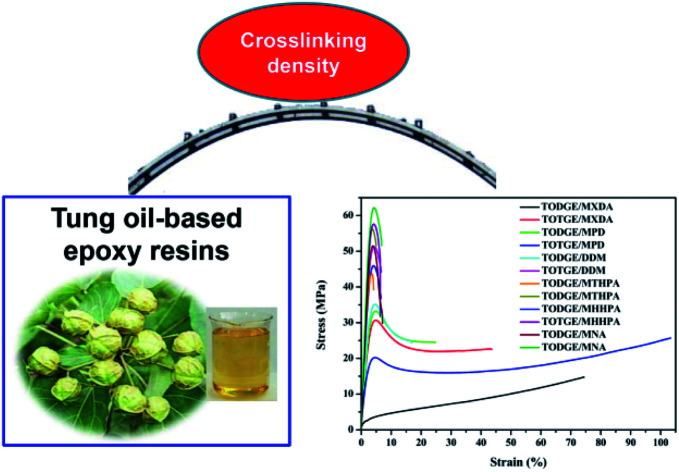

## 1. Introduction

Epoxy resins have been adopted in various industrial applications like adhesives, automobiles, and aerospace because of their excellent properties, such as excellent adhesive strength, tunable mechanical properties, good dimensional stability, and chemical resistance.^1–10^ However, almost all of the epoxy resins are from fossil resources. Concerning the environmental impact of petroleum-based epoxy resin and depletion of non-renewable resources, the exploitation of bio-based epoxy resin has attracted a lot of attention.^11–14^

**Fig. 1:**
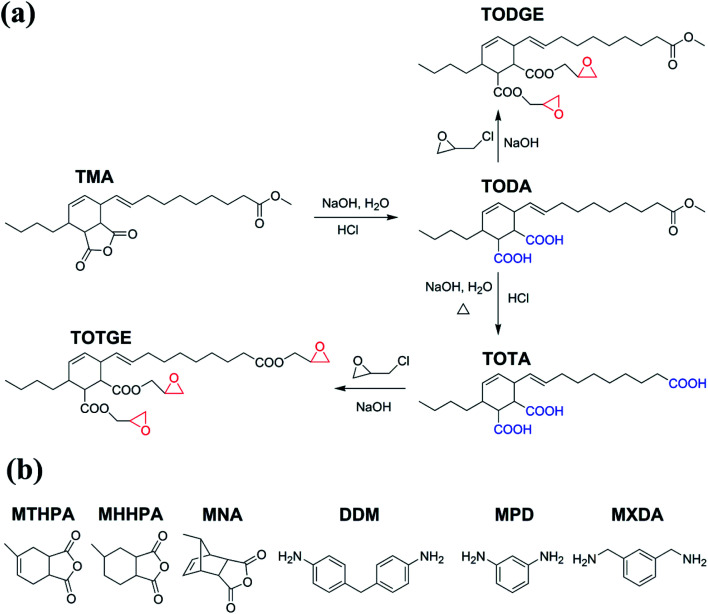
Synthetic routes of TODGE and TOTGE and the chemical structures of curing agents used in this study.

Plant oils are major agricultural commodities in the world.^15^ Recently, they have been applied to synthesize bio-based epoxy resin due to their low price, wide availability, inherent biodegradability, and low toxicity.^11–14^ Epoxidized plant oils are major plant oil-based epoxy resins. However, these plant oil-based epoxy resins always show low mechanical and thermal properties, because of their low reactive epoxy groups and long fatty chains.^16^ To overcome these drawbacks, tung oil-based epoxy resin TGEC22, which had comparable mechanical and thermal properties to commercial bisphenol A epoxy resin DER332, was fabricated by introducing highly reactive terminal epoxy groups and high crosslinking density.^17^ However, the TGEC22 showed severe brittleness as the DER332 because of its high crosslinking density. Decreasing the crosslinking density by reducing the epoxy groups resulted in a significant reduction of strength, modulus, and thermal properties, but a negligible improvement in toughness.^17^ It is well known that the crosslinking density plays an important role in the performance of epoxy resin.^16–30^ However, so far there is no systematic study about the effect of the crosslinking density on the properties of plant oil-based epoxy resins. Understanding the relationship between the crosslinking density and the properties of plant oil-based epoxy resins is essential to design plant oil-based epoxy resins. To address this issue, model tung oil-based diglycidyl ester (TODGE) and tung oil-based triglycidyl ester (TOTGE) were synthesized and cured with different hardeners to prepare tung oil-based epoxy resins with different crosslinking densities, as shown in Fig. 1. The mechanical and thermal properties of the cured resins were studied systematically to establish the relationship between the crosslinking density and the thermo-mechanical properties of tung oil-based epoxy resins. It is found that linear increases are observed between the tensile strength, Young's modulus, and glass transition temperature and the crosslinking density. The elongation at break and tensile toughness have nonlinear relationships with the crosslinking density. No direct relationship is observed between the thermostability and the crosslinking density because the thermostability is determined by the molecular structure of curing system and the crosslinking density.

## 2. Experimental section

### 2.1 Materials

Adduct of methyl esters of tung oil fatty acids and maleic anhydride (TMA) was obtained from the Institute of Chemical Industry of Forestry Products. Methyl tetrahydrophthalic anhydride (MTHPA, 99%), methyl nadic anhydride (MNA, 99%), 4,4′-diaminodiphenyl methane (DDM, 99%), *m*-phenylenediamine (MPD, 99%), *m*-xylylenediamine (MXDA, 99%), 2,4,6-tris(dimethylaminomethyl)phenol (DMP-30, 95%), and benzyltriethylammonium chloride (98%) were purchased from Shanghai Aladdin Bio-Chem Technology Co., Ltd. Hydrochloric acid (HCl, 36∼38 wt%), sodium hydroxide (NaOH, 99%), ethyl acetate (99%), ethanol (99%), and calcium oxide (CaO, 99%) were received from Nanjing Chemical Reagent Co., Ltd. Epichlorohydrin (99%) was obtained from Shanghai Lingfeng Chemical Reagent Co., Ltd.

### 2.2 Synthesis

#### 2.2.1 Synthesis of tung oil-based dicarboxylic acid (TODA) and TODGE

Crude TMA (390 g) mixed with 20 wt% NaOH aqueous solution (the mass of NaOH was 40 g) at room temperature for 2 h. Then, a certain amount of distilled water and ethyl acetate were added to separate methyl esters of fatty acids impurities. The water layer was acidified using a 1 M HCl solution until the pH value was 2–3. After that, ethyl acetate was added and washed with H_2_O until the pH of H_2_O was 7. The organic layer was collected and the ethyl acetate was removed by rotary evaporation under vacuum to obtain 348.86 g TODA (yield: 89.45% with respect to the crude TMA). The acid value of TODA was 268 mg (KOH) per g (theory: 275 mg (KOH) per g).

TODA (40.8 g, 0.1 mol), epichlorohydrin (185 g, 2 mol), and benzyltriethyl ammonium chloride (0.46 g, 0.002 mol) were charged into 500 ml flask and reacted at 117 °C for 4 h. After the mixture was cooled to 60 °C, 8 g NaOH (0.2 mol) and 11.2 g CaO (0.2 mol) were added and stirred at 60 °C for 3 h. Then, the mixture was filtered and unreacted epichlorohydrin was removed by a rotary evaporator under vacuum to obtain 43 g of a bright yellowish viscous TODGE. The yield of TODGE was 83% relative to theory and the epoxide value of TODGE was 0.35 equiv./100 g (theory: 0.38 equiv./100 g).

#### 2.2.2 Synthesis of tung oil-based tricarboxylic acid (TOTA) and TOTGE

NaOH (25 g) and H_2_O (60 g) were charged into 250 ml flask and heated to 80 °C. Then, TODA/ethanol solution (40.8 g TODA was dissolved in 40 ml ethanol) was dropped into the NaOH solution. After that, the solution was stirred at 90 °C for 4 h. When the solution was cooled to 60 °C, the solution was acidified using a 1 M HCl solution. The organic layer was collected and then mixed with ethyl acetate. The solution was washed with H_2_O until the pH of H_2_O was 7. The reddish-brown TOTA (37.2 g) was obtained by removing the ethyl acetate and water *via* rotary evaporation under vacuum (39.4 g in theory, yield: 94%). The acid value of TOTA was 381 mg (KOH) per g (theory: 427 mg (KOH) per g).

The synthesis of TOTGE was similar to that of the TODGE. The epoxide value of TOTGE was 0.43 equiv./100 g (theory: 0.53 equiv./100 g) and the yield was 89%.

### 2.3 Preparation of epoxy samples

The tung oil-based glycidyl ester (TODGE or TOTGE) mixed with a stoichiometric amount of curing agent at room temperature. For anhydride curing agent, DMP-30 as a catalyst was added and the amount was 0.5 wt% of the total weight of the curing agent and tung oil-based glycidyl ester. The mixtures were degassed and poured into the stainless steel molds for curing. For anhydride curing agent, the mixtures were cured at 100 °C for 2 h, 120 °C for 2 h, and 150 °C for 2 h. For the selective amine curing agents, the mixtures were cured at room temperature for 24 h and 80 °C for 4 h.

### 2.4 Characterization

#### 2.4.1 Structural characterization

Fourier transform infrared (FTIR) analysis was performed with a Thermo Scientific Nicolet iS10 spectrometer. The ^1^H nuclear magnetic resonance (^1^H NMR) spectra of the compounds were obtained from a Bruker 400 MHz spectrometer. The NMR test was conducted at room temperature and the solvent was deuterated chloroform (CDCl_3_) or deuterated dimethyl sulfoxide (DMSO).

#### 2.4.2 Measurement of mechanical properties

Tensile testing was performed by using an Instron 4201 machine equipped with a 1 kN electronic load cell. The tests were performed at a 5 mm min^−1^ crosshead speed at 25 °C. At least five specimens of each sample were tested and average values were reported. The toughness of each sample was obtained by integrating the area under the stress–strain curves.

### 2.5 Measurement of thermal properties

Dynamic mechanical analysis (DMA) was performed using a Q800 dynamic mechanical analyzer (TA Instruments) in a dual cantilever mode with an oscillating frequency of 1 Hz. The temperature ranged from −50 to 150 °C and the heating rate was 3 °C min^−1^. The glass transition temperature (*T*_g_) can be obtained from the curve of the loss factor tan *δ versus* temperature. The crosslinking density (*v*_c_) of the cured tung oil-based epoxy resins was calculated from the following formula:^17,23^1
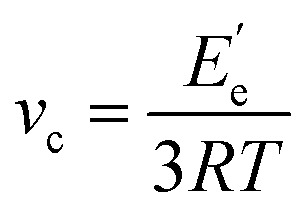
where *R* is the gas constant, *T* is the absolute temperature at *T*_g_ + 50 °C, and 
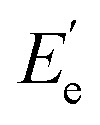
 is the storage modulus at *T*_g_ + 50 °C.

The thermostability of the cured tung oil-based epoxy resin was detected by thermogravimetric analysis (TGA), which was performed on a TG209F1 TGA (Netzsch) instrument. Each sample was scanned from 35 °C to 800 °C at a heating rate of 10 °C min^−1^ under a nitrogen atmosphere.

## 3. Results and discussion

### 3.1 Synthesis and characterizations of model tung oil-based epoxy resins

**Fig. 2:**
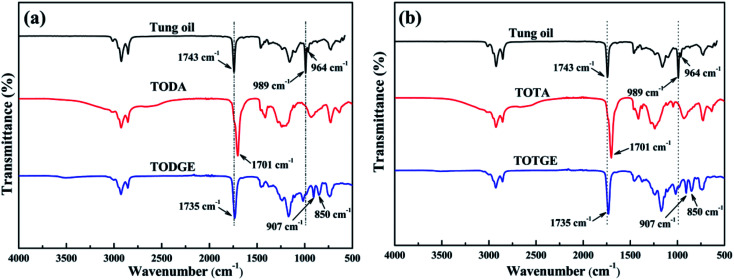
FTIR spectra of tung oil, TODA, TODGE, TOTA, and TOTGE.

**Fig. 3:**
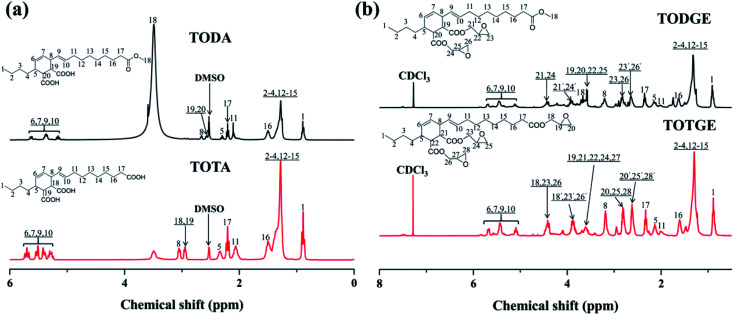
^1^H NMR spectra of (a) TODA and TOTA, (b) TODGE and TOTGE.

Tung oil-based epoxy resins are synthesized from industrial product TMA (Fig. 1), which is the adduct of methyl esters of tung oil fatty acids and maleic anhydride. Therefore, for the FTIR spectra of intermediates (TODA and TOTA) and tung oil-based epoxy resins (TODGE and TOTGE), the conjugated diene group (989 cm^−1^ and 964 cm^−1^) is disappeared, as shown in Fig. 2. In this study, the tung oil-based epoxy resin is obtained from the reaction between the carboxyl group and the epoxy group. Therefore, after the reaction, the carboxyl group (1701 cm^−1^) is disappeared and new groups including the ester group (1735 cm^−1^) and the epoxy group (907 cm^−1^ and 850 cm^−1^) are appeared. The change of the molecular structure is also evident from the ^1^H NMR spectra (Fig. 3). The attributions of the protons' chemical shifts of TODA, TOTA, TODGE, and TOTGE are labeled in the Fig. 3. For TODA, the chemical shift at 3.59 ppm is attributed to the protons of the terminal CH_3_ group attached with ester bond (Fig. 3a). After saponification and acidification, the protons of the terminal CH_3_ group attached with ester bond (3.59 ppm) is almost disappeared, indicating a carboxyl group is formed in the TOTA (Fig. 3a). Fig. 3b is the ^1^H NMR spectra of TODGE and TOTGE. The protons of the glycidyl ester of TODGE exhibit chemical shifts between 2.64 and 4.44 ppm and that of TOTGE between 2.62 and 4.41 ppm.^17^ The results of FTIR spectra and ^1^H-NMR analyses prove the successful synthesis of TODGE and TOTGE.

### 3.2 The properties of tung oil-based epoxy resins

#### 3.2.1 Dynamic mechanical analysis

**Fig. 4:**
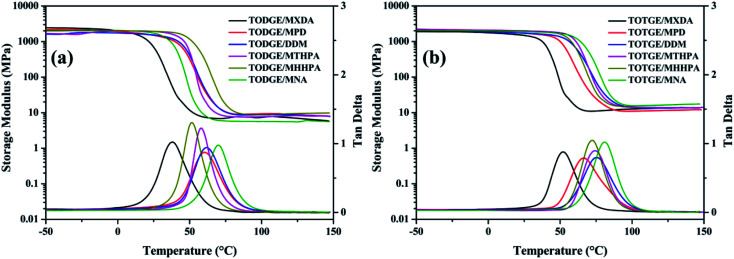
Storage modulus and tan delta of (a) TODGE and (b) TOTGE curing with different curing agents.

Fig. 4 shows the temperature dependences of storage modulus (*E*′) and loss factor (tan delta) of tung oil-based epoxy resins curing with different curing agents. From the tan delta *versus* temperature curves, *T*_g_ of the cured tung oil-based epoxy resin is obtained and listed in the Table 1. The *T*_g_ is ranged from 37.7 to 81.0 °C, which may be attributed to different crosslinking densities. The crosslinking densities of the cured tung oil-based epoxy resins are calculated from the storage modulus–temperature curves according to eqn (1) and presented in Table 1. From the table, it can be obtained that the crosslinking density has a devastating effect on the *T*_g_.

**Table 1:** DMA parameters of tung oil-based epoxy resins curing with different curing agents

Sample	*T*_g_ (°C)	*T*_g_ + 50 °C	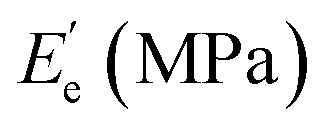	*v*_c_ (10^−3^ mol cm^−3^)
TODGE/MXDA	37.7	87.7	7.57	0.84
TOTGE/MXDA	52.0	102.0	12.73	1.36
TODGE/MPD	60.4	110.4	9.28	0.97
TOTGE/MPD	66.5	116.5	11.34	1.17
TODGE/DDM	61.7	111.7	8.89	0.93
TOTGE/DDM	75.1	125.1	13.52	1.36
TODGE/MTHPA	57.7	107.7	7.86	0.83
TOTGE/MTHPA	73.8	123.8	13.88	1.40
TODGE/MHHPA	51.3	101.3	5.69	0.61
TOTGE/MHHPA	72.1	122.1	16.23	1.65
TODGE/MNA	69.9	119.9	9.34	0.95
TOTGE/MNA	81.0	131.0	16.69	1.66

#### 3.2.2 Mechanical properties

**Fig. 5:**
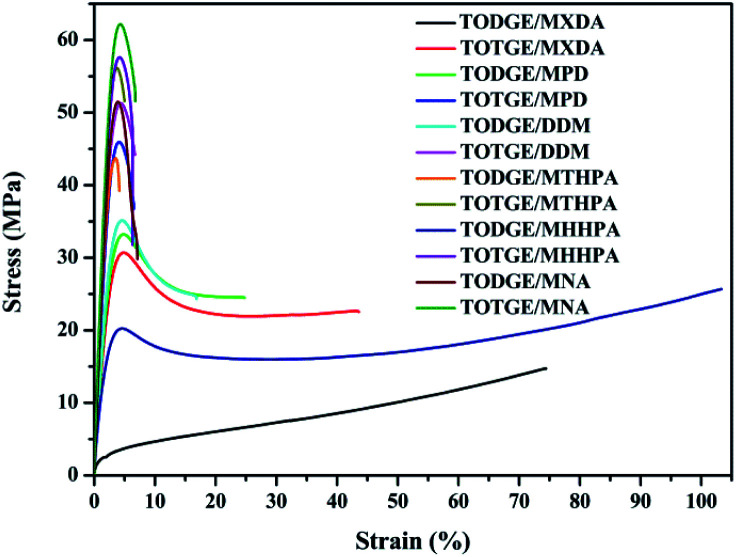
Representative stress–strain curves of tung oil-based epoxy resins curing with different curing agents.

Fig. 5 shows representative stress–strain curves of different hardeners cured tung oil-based epoxy resins. The mechanical properties are summarized in Table 2. From Fig. 5 and Table 2, it is clear that higher tensile strength and Young's modulus, lower elongation at break and toughness are observed for the cured TOTGE compared to the cured TODGE curing with the same curing agent. This is because the TOTGE has more epoxy functional groups than that of the TODGE, which results in higher crosslinking density achieved for the TOTGE. From Fig. 5 and Table 2, it is obtained that the mechanical properties have a relationship with the crosslinking density.

**Table 2:** Mechanical properties of tung oil-based epoxy resins curing with different curing agents

Sample	Tensile strength (MPa)	Elongation at break (%)	Young's modulus (MPa)	Toughness^a^ (MJ m^−3^)
TODGE/MXDA	14.22 ± 1.79	74.03 ± 8.96	379.86 ± 67.97	6.07 ± 1.12
TOTGE/MXDA	31.18 ± 0.56	41.84 ± 2.44	1510.66 ± 80.55	6.56 ± 3.63
TODGE/MPD	33.52 ± 0.63	23.50 ± 5.82	1527.60 ± 27.54	6.20 ± 1.37
TOTGE/MPD	45.40 ± 0.86	6.92 ± 1.71	1987.79 ± 58.12	2.43 ± 0.65
TODGE/DDM	35.62 ± 0.78	12.87 ± 4.09	1555.02 ± 40.20	3.59 ± 1.11
TOTGE/DDM	52.48 ± 2.02	6.47 ± 0.45	2368.17 ± 114.46	2.65 ± 0.04
TODGE/MTHPA	43.38 ± 2.05	4.14 ± 0.96	2205.40 ± 114.91	1.32 ± 0.27
TOTGE/MTHPA	56.19 ± 1.31	3.75 ± 1.70	2662.37 ± 51.39	1.40 ± 0.87
TODGE/MHHPA	23.72 ± 2.78	100.79 ± 3.61	1298.65 ± 8.08	18.14 ± 1.69
TOTGE/MHHPA	58.35 ± 3.13	5.50 ± 0.51	2507.47 ± 143.39	2.35 ± 0.30
TODGE/MNA	51.69 ± 0.65	6.17 ± 1.33	2324.22 ± 196.42	2.34 ± 0.45
TOTGE/MNA	62.61 ± 0.42	6.68 ± 0.11	2560.22 ± 40.47	3.23 ± 0.06

a The value is calculated by integrating the area under stress–strain curves.

#### 3.2.3 Thermogravimetric analysis

**Fig. 6:**
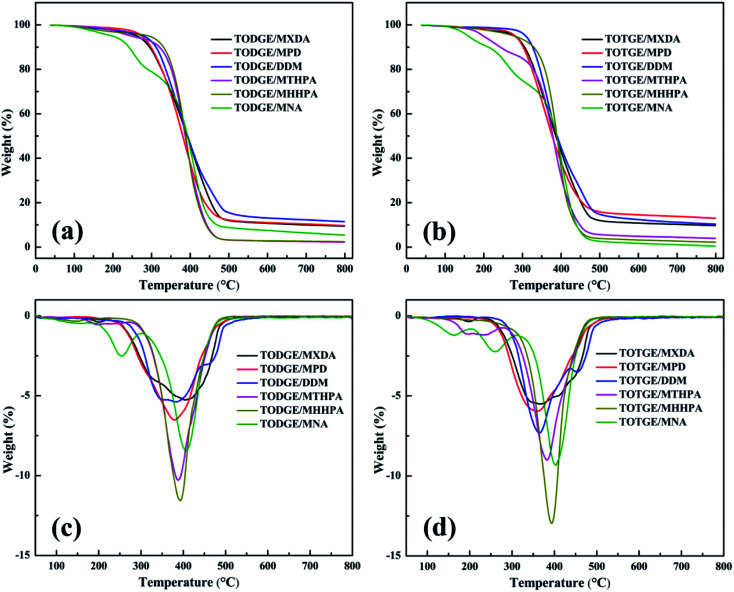
TGA results of cured tung oil-based epoxy resins: (a) thermogravimetric curves of the cured TODGE, (b) thermogravimetric curves of the cured TOTGE, (c) derivative thermogravimetric curves of the cured TODGE, and (d) derivative thermogravimetric curves of the cured TOTGE.

TGA can detect the thermostability of the cured tung oil-based epoxy resins. Fig. 6 shows the TGA results of the tung oil-based epoxy resins cured with different hardeners. Table 3 summarizes characteristic parameters of thermal properties of the cured tung oil-based epoxy resins. For curing agents MTHPA and MNA, the cured tung oil-based epoxy resins experience two-stage thermal decompositions. The first stage of decomposition is attributed to the decomposition of small molecules,^31^ which may be from the reverse Diels–Alder reactions of MTHPA and MNA at high temperature.^32^ Then, the decomposition of the relatively stable tung oil-based epoxy cross-linked network contributes to the second stage of decomposition. However, for other hardeners cured tung oil-based epoxy systems, only one pyrogenation process is observed, which may be due to the presence of more thermally stable structures for other curing agents.^33^ From Table 3, it is clear that the thermal stability of the cured TOTGE epoxy resins is almost much higher than that of the same curing agent cured TODGE epoxy resins due to their high higher crosslinking densities for the cured TOTGE epoxy resins with the same hardener. This indicates that the crosslinking density has an effect on the thermal stability.

**Table 3:** Characteristic thermostability of tung oil-based epoxy resins curing with different curing agents

Sample	*T*_10%_ a (°C)	*T*_m_ b (°C)	Char residue at 800 °C (%)
TODGE/MXDA	291.3 ± 3.5	411.4 ± 9.8	8.42 ± 1.44
TOTGE/MXDA	305.0 ± 1.8	398.0 ± 45.6	9.27 ± 0.62
TODGE/MPD	296.2 ± 3.4	362.0 ± 24.8	11.69 ± 2.86
TOTGE/MPD	300.1 ± 1.8	379.7 ± 27.6	12.66 ± 0.52
TODGE/DDM	311.2 ± 0.2	363.9 ± 29.4	12.75 ± 1.82
TOTGE/DDM	324.8 ± 2.2	388.0 ± 30.1	12.03 ± 2.38
TODGE/MTHPA	326.4 ± 7.3	389.1 ± 1.9	1.16 ± 1.59
TOTGE/MTHPA	350.5 ± 12.4	397.0 ± 7.7	2.97 ± 1.19
TODGE/MHHPA	331.5 ± 3.6	393.7 ± 0.5	2.72 ± 0.41
TOTGE/MHHPA	332.7 ± 1.7	394.2 ± 1.2	1.85 ± 0.54
TODGE/MNA	257.9 ± 22.8	408.8 ± 2.4	5.30 ± 0.18
TOTGE/MNA	261.2 ± 3.1	406.8 ± 1.2	5.61 ± 1.85

a *T*_10%_ is the temperature corresponding to 10% mass loss, which represents the temperature of degradation.^23,34^

b *T*_m_ is the temperature of the maximum decomposition rate.

### 3.3 Structure–property relationship

**Fig. 7:**
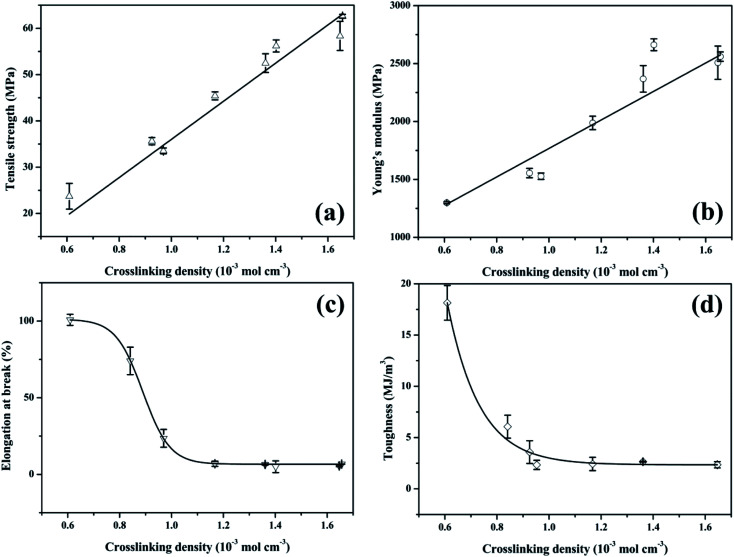
The mechanical properties of cured tung oil-based epoxy resins as a function of crosslinking density: (a) tensile strength, (b) Young's modulus, (c) elongation at break, and (d) tensile toughness.

**Fig. 8:**
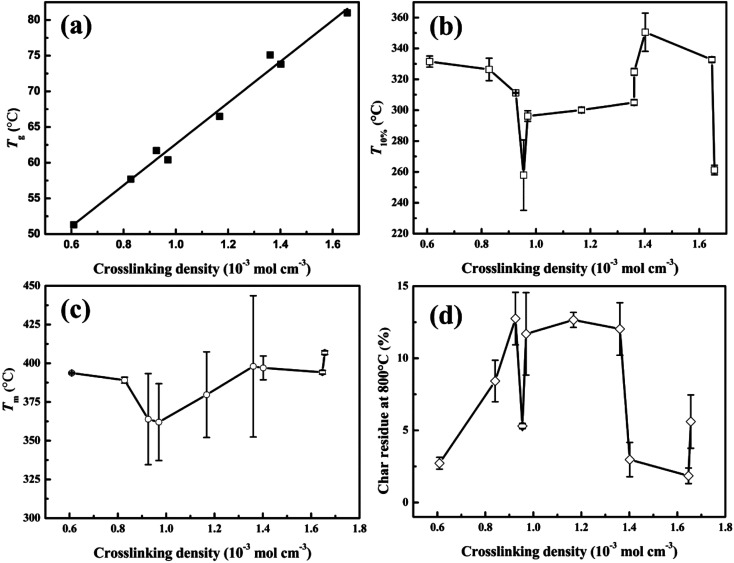
The thermal properties of cured tung oil-based epoxy resins as a function of crosslinking density: (a) *T*_g_, (b) *T*_i_, (c) *T*_m_, and (d) char residue at 800 °C.

From the aforementioned statements, it is clear that the crosslinking density plays an important role in the properties of tung oil-based epoxy resins. To understand the relationship between the crosslinking density and the thermo-mechanical properties of plant oil-based epoxy resins, the plots of mechanical and thermal properties *versus* crosslinking density were charted, as shown in Fig. 7 and 8.

Fig. 7 presents the plot of the mechanical properties of the tung oil-based epoxy resins *versus* crosslinking density. For tensile strength *σ*_b_, previous studies reported that the crosslinking density of epoxy resins had a strong influence on the tensile strength.^35–37^ For the cured tung oil-based epoxy resins in our study, the tensile strength shows a linear increase with increasing the crosslinking density (Fig. 7a), which is consistent with previous study.^37^ This linear increase of the tensile strength is fitted to the function: *σ*_b_ = −5.21 + 41.21*v*_c_. For Young's modulus *E*, some studies showed that the crosslinking density had a weak effect on the Young's modulus of epoxy resin.^35,36^ Others showed that the Young's modulus of epoxy resin was significantly affected by the crosslinking density.^21,37–39^ Herein, the influence of the crosslinking density on the Young's modulus of tung oil-based epoxy resin is studied, as shown in Fig. 7b. The Young's modulus shows a similar trend as the tensile strength, which is in good agreement with previous observations.^37–39^ This linear increase of the Young's modulus is fitted to the function: *E* = 1229.98*v*_c_ + 537.39. For elongation at break *ε*_b_, it decreases slowly at first, then dramatically, and finally gently as the crosslinking density increases (Fig. 7c). This nonlinear decrease of the elongation at break is fitted to the Boltzmann function: *ε*_b_ = 6.62 + 94.59/(1 + e^(*v*_c_−0.89)/0.05^). The tensile toughness *T* also displays a nonlinear decrease. The toughness decreases dramatically at first and then slowly with increasing the crosslinking density (Fig. 7d). This nonlinear decrease of the tensile toughness is fitted to exponential function: *T* = 2220.42 e^(−*v*_c_/0.12)^ + 2.35. Recently, Wang found that the toughness of epoxidized plant oil was enhanced with increasing the crosslinking density due to the existence of phase separation,^26^ which is conflicted with our observation. For the tung oil-based epoxy curing system, no phase separation is observed because the tensile failure surface is homogeneous (Fig. S1†). Therefore, for the tung oil-based epoxy curing system, phase separation toughening phenomenon is not occurred and the toughness is decreased as the crosslinking density increases.

Fig. 8 presents the plot of the thermal properties of the tung oil-based epoxy resins *vs.* crosslinking density. Fig. 8a shows the relationship between *T*_g_ and the crosslinking density. It is clear that *T*_g_ increases linearly with increasing the crosslinking density. This behaviour is similar to that observed by previous studies.^16,18,35–37^ This *T*_g_ increase is fitted to linear function: *T*_g_ = 28.87*v*_c_ + 33.75. From Fig. 8b–d, it is found that the crosslinking density has no function with the characteristic parameters of the thermostability. For the initial decomposition temperature *T*_10%_ (Fig. 8b), *T*_10%_ decreases as the crosslinking density increases when the crosslinking density is lower than 0.90 mol dm^−3^. After that, *T*_10%_ increases with increasing the crosslinking density. And then, *T*_10%_ decreases with increasing the crosslinking density. When the crosslinking density is 0.95 and 1.66 mol dm^−3^ (the curing systems are TODGE/MNA and TOTGE/MNA), a sharp reduction of *T*_10%_ is observed because low-molecular-weight components are generated when the reverse Diels–Alder reaction of MNA is happened at high temperature.^31,32^Fig. 8c shows the variation trend of the maximum decomposition temperature *T*_m_ with the enhancement of the crosslinking density. For low crosslinking density (<1.0 mol dm^−3^), *T*_m_ shows a downward trend as the crosslinking density increases. For high crosslinking density (>1.0 mol dm^−3^), a rising trend is presented. For char residue at 800 °C (Fig. 8d), it shows a upward, and then a downward, and finally a upward trend as an increase in the crosslinking density. It should be noted that the curing system with aromatic structure has higher char yield than that with aliphatic structure, because the char yield is inversely proportional to the hydrogen content of the polymers.^40^ As a result, the char residue shows a rise variation trend at first (the crosslinking density is lower than 1.4 mol dm^−3^) apart from the char residue at the crosslinking density of 0.95 mol dm^−3^ (the curing system is TODGE/MNA), because almost all curing agents have aromatic structure at this crosslinking density range. When the crosslinking density is 0.61 mol dm^−3^, the curing system is TODGE/MHHPA, which doesn't possess aromatic structure. As the crosslinking density increases to 0.84 mol dm^−3^, the curing system (TODGE/MXDA) has aromatic structure. The char residue of TODGE/MXDA curing system shows a sharp increase due to the enhancement of crosslinking density and possessing aromatic structure. When the crosslinking density is 0.95 mol dm^−3^, a sharp drop is observed because no aromatic structure is existed in the TODGE/MNA curing system. When the crosslinking density is over 1.36 mol dm^−3^, all curing systems contain no aromatic structure. As a result, the char yield show a sharp drop and then a rise as the crosslinking density increases. From these analyses, it can be obtained that the thermostability is determined by both the structure of curing system and the crosslinking density.^41^

## 4. Conclusions

Tung oil-based epoxy resins with different crosslinking densities were fabricated from curing tung oil-based glycidyl esters with different hardeners. The mechanical and thermal properties of tung oil-based epoxy resins with different crosslinking densities were systematically studied to establish the relationship between the crosslinking density and the thermo-mechanical properties. It was found that the tensile strength, Young's modulus, and glass transition temperature had linear relationships with the crosslinking density. They were linearly increased as the crosslinking density increased. The elongation at break and tensile toughness had nonlinear relationships with the crosslinking density. The elongation at break decreased gently at first, then dramatically, and finally slowly as the crosslinking density increased. The tensile toughness declined sharply at first and then slowly with increasing the crosslinking density. No direct relationship was observed between the thermostability and the crosslinking density because the thermostability was determined by both the molecular structure of curing system and the crosslinking density.

## Conflicts of interest

There are no conflicts to declare.

## Supplementary Material

RA-012-D2RA04206A-s001
