# Supplementary material for: Influence of crosslinking density on the mechanical and thermal properties of plant oil-based epoxy resin
Source: RSC Adv. 2022 Aug 16;12(36):23048–56. doi: 10.1039/d2ra04206a (PMC9379777; doi:10.1039/d2ra04206a)
Supplement: RA-012-D2RA04206A-s001 [file RA-012-D2RA04206A-s001.pdf]

Electronic Supplementary Material (ESI) for RSC Advances.
This journal is © The Royal Society of Chemistry 2022

Electronic Supplementary Information

Influence of crosslinking density on the mechanical and thermal properties of plant oil-based epoxy resin

Jinrui Huang[1, 2*], Pan Fu[2], Wenbin Li[2], Laihui Xiao[2], Jie Chen[2], Xiaoan Nie[2*]

[1] Institute of Ecological Conservation and Restoration, Chinese Academy of Forestry, Beijing 100091, China

[2] Key Laboratory of Biomass Energy and Material, Jiangsu Province; Jiangsu Co-Innovation Center of Efficient Processing and Utilization of Forest Resources; Key Laboratory of Chemical Engineering of Forest Products, National Forestry and Grassland Administration; National Engineering Research Center for Low-Carbon Processing and Utilization of Forest Biomass; Institute of Chemical Industry of Forest Products, Chinese Academy of Forestry, Nanjing 210042, Jiangsu Province, China

***Corresponding Authors**

E-mail: huangjinruiahu@126.com; jrhuang@caf.ac.cn (J. R. Huang).

E-mail: niexiaoan@icifp.cn (X. A. Nie).

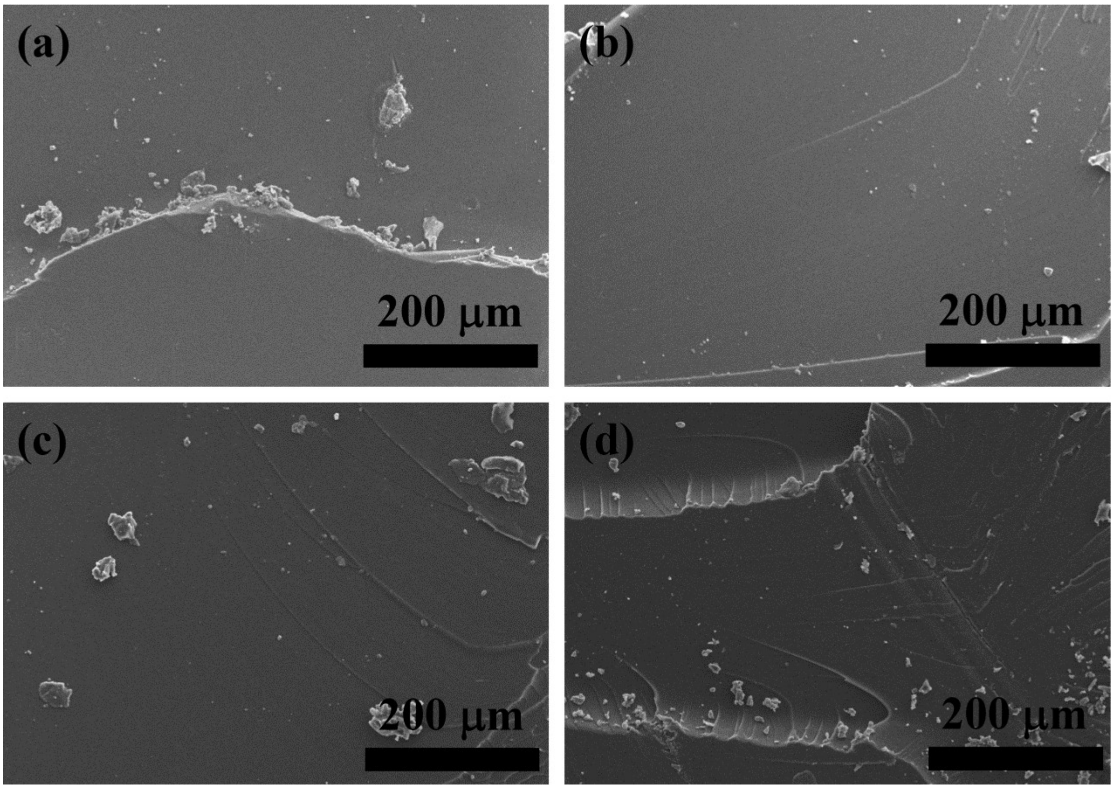

Fig. S1 SEM images of tung oil-based epoxy resins curing with different curing agents after tensile tests: (a) TODGE-MHHPA, (b) TODGE-DDM, (c) TOTGE-MPD, and (d) TOTGE-DDM.
